# Peripheral Organ Equivalent Dose Estimation Procedure in Proton Therapy

**DOI:** 10.3389/fonc.2022.882476

**Published:** 2022-05-25

**Authors:** Carles Domingo, Juan Ignacio Lagares, Maite Romero-Expósito, Beatriz Sánchez-Nieto, Jaime J. Nieto-Camero, Jose Antonio Terrón, Leticia Irazola, Alexandru Dasu, Francisco Sánchez-Doblado

**Affiliations:** ^1^ Departament de Fisica, Universitat Autònoma de Barcelona, Bellaterra, Spain; ^2^ Unidad de Aplicaciones Médicas, Departamento de Tecnología, Centro de Investigaciones Energéticas Medioambientales y Tecnológicas (CIEMAT), Madrid, Spain; ^3^ The Skandion Clinic, Uppsala, Sweden; ^4^ Instituto de Física, Pontificia Universidad Católica de Chile, Santiago, Chile; ^5^ Medical Radiation Group, iThemba Labs, Faure, South Africa; ^6^ Servicio de Radiofísica, Hospital Universitario Virgen Macarena, Sevilla, Spain; ^7^ Servicio de Radiofísica y Protección Radiológica, Clínica Universidad de Navarra, Pamplona, Spain; ^8^ Medical Radiation Sciences, Department of Immunology, Genetics and Pathology, Uppsala University, Uppsala, Sweden; ^9^ Dpto Fisiología Médica y Biofísica, Universidad de Sevilla, Sevilla, Spain

**Keywords:** peripheral organ dose, proton therapy, neutron spectrometry, secondary cancer risk, neutron and photon dose

## Abstract

The aim of this work is to present a reproducible methodology for the evaluation of total equivalent doses in organs during proton therapy facilities. The methodology is based on measuring the dose equivalent in representative locations inside an anthropomorphic phantom where photon and neutron dosimeters were inserted. The Monte Carlo simulation was needed for obtaining neutron energy distribution inside the phantom. The methodology was implemented for a head irradiation case in the passive proton beam of iThemba Labs (South Africa). Thermoluminescent dosimeter (TLD)-600 and TLD-700 pairs were used as dosimeters inside the phantom and GEANT code for simulations. In addition, Bonner sphere spectrometry was performed inside the treatment room to obtain the neutron spectra, some relevant neutron dosimetric quantities per treatment Gy, and a percentual distribution of neutron fluence and ambient dose equivalent in four energy groups, at two locations. The neutron spectrum at one of those locations was also simulated so that a reasonable agreement between simulation and measurement allowed a validation of the simulation. Results showed that the total out-of-field dose equivalent inside the phantom ranged from 1.4 to 0.28 mSv/Gy, mainly due to the neutron contribution and with a small contribution from photons, 10% on average. The order of magnitude of the equivalent dose in organs was similar, displaying a slow reduction in values as the organ is farther from the target volume. These values were in agreement with those found by other authors in other passive beam facilities under similar irradiation and measurement conditions.

## 1 Introduction

Worldwide, an estimated 19.3 million new cancer cases were diagnosed in 2020 (1). Many of these cancers can be cured if detected early and treated efficiently. Noteworthy, more than 50% of the diagnosed patients undergo radiotherapy (RT), alone or in combination with chemotherapy or surgery, at some stage of the treatment. Photon RT techniques are the most common, and they have progressed very efficiently, from the geometrical conformation of fields to modulated intensity and RapidArc treatments, so that their therapeutic potential has increased. Nonetheless, this benefit has been accompanied by a growing concern about the risk of second radiation-induced tumors. It has been long known that patients treated with ionizing radiation carry a risk of developing a second cancer in their lifetimes, but the renewed concern comes from the substantial improvements in cancer survival, longer than the latency time of second cancers, together with the potential increase of out-of-field doses to healthy tissues distant from the target volume, which might be more significant for the intensity-modulated techniques (2, 3).

Compared with photon RT, proton therapy has the benefit of achieving up to 60% reduction of the radiation dose delivered to the healthy tissues around the tumor (3–5) while delivering a higher dose to the tumor itself. Therefore, proton therapy appeared as a safer and more effective therapy for some anatomic sites and tumors than photon therapy. The rationale is that, due to the Bragg peak, proton therapy provides a lower radiation dose to the non-target tissue while a high dose is delivered to a very specific area. These results are based on the physics fact that the proton range in tissue is finite while photon absorption follows an exponential decay function, and hence, some doses are received for the full-beam path in the body. However, the absorbed dose is not everything; it is necessary to consider the relative biological effectiveness (RBE) of the different out-of-field particles. Precisely, the higher RBE of neutrons compared to photons, the larger the biological impact of the former. In photon RT, the main contribution to out-of-field doses comes from stray photons, with a smaller contribution from neutrons when high energies are used for irradiation. On the contrary, neutrons are the main contributors to out-of-field doses in proton therapy. Despite this, overall proton therapy generally offers a substantial benefit in the non-target dose, as the out-of-field equivalent dose resulting from proton therapy is typically smaller than that resulting from photon RT (6). Consequently, it has been claimed that the second cancer risk associated with proton therapy is lower than that expected in photon therapy (7). This advantage is more obvious for low-energy proton treatments and scanning beam therapy (6), being one of the arguments toward the current tendency to the clinical use of scanning vs. passive proton beam equipment. However, tens of thousands of patients were already (and are currently) treated with passive scattering beams, which cannot be disregarded as they are an invaluable data source in terms of longer clinical follow-up for epidemiological studies (8).

Neutron dosimetry is very challenging, particularly the estimation of the neutron equivalent dose to organs in patients under proton therapy. Finding a methodology for this issue would optimize future proton treatments and bring out epidemiology studies to develop more accurate cancer risk prediction models (9).

The goal of this work was to establish a methodology for evaluating the peripheral neutron and photon equivalent dose to organs at risk, valid for any situation in proton therapy. The authors have experience in determining peripheral neutron and photon equivalent doses in organs for photon RT (10–17). The steps already followed for the implementation of the procedure in photon RT were applied in this work to proton therapy. Firstly, neutron spectra measurements were made at specific points inside the treatment room using an extended-range Bonner sphere spectrometer (ERBSS). A Monte Carlo (MC) simulation of the neutron field in the treatment room and inside an anthropo-geometrical phantom was performed and validated with the ERBSS measurements. Secondly, measurements inside the phantom using photon and neutron dosimeters were performed with calibrated passive dosimeters. The above data allowed the calculation of the equivalent dose in the patient’s organs, which is the relevant quantity for estimating a second cancer risk. Measurements and simulations were performed for the iThemba proton therapy facility (Cape Town, South Africa).

## 2 Material and Methods

### 2.1 Irradiations

**Figure 1:**
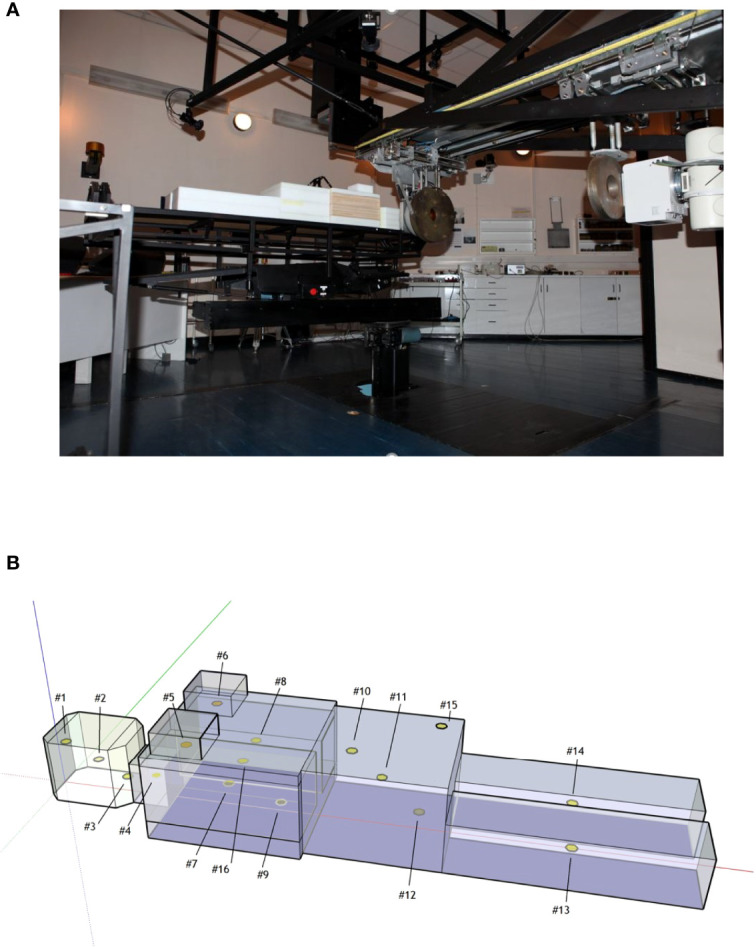
**(A)** NORMA phantom during the head irradiation in the iThemba facility. **(B)** Sketch of NORMA phantom and detector positions.

The adult female anthropomorphic phantom (NORMA) (**Figure 1**) (11) was used for irradiations and was modeled for simulations. This phantom was manufactured in polyethylene, except for the low-density wood that was used for simulating lung tissue. This material composition was previously validated as adequate for mimicking neutron interaction with human tissue (17). Sixteen customized detector holes (see **Figure 1** and **Table 1**) were distributed inside NORMA, at different positions and depths, so that the detectors placed in them could be used to determine the equivalent dose in relevant organs.

**Table 1:** Points in NORMA phantom.

Point	Location	Distance to iso* in CC direction (cm)
1	Head up	9
2	Head medium	0
3	Head down	8
4	Neck	17
5	Right breast	31
6	Left breast	31
7	Right thorax lung	42
8	Left thorax lung	42
9	Thorax spine	52
10	Pelvis up	71
11	Pelvis medium	79
12	Pelvis down	89
13	Right leg	127
14	Left leg	127
15	Skin	92
16	Mediastine	42

*In the head treatment. CC, craneo-caudal.

The iThemba proton therapy facility uses a 200 MeV fixed horizontal beam line with collimator arrangements and energy degraders to properly define the irradiated volume (8). In our case, two types of static field treatments were considered:

- In the pelvic region, with an irradiated cylindrical volume 3 cm in diameter and 3 cm height centered at the pelvis mid-point, identified as point#11 in the NORMA phantom (11). Neutron spectrometry, as described in Section *Bonner Sphere Spectrometry* was performed in two positions inside the treatment room during this irradiation with the aim of validating the MC simulation.
- In the head region, reproducing a brain treatment, in an irradiated volume equal to that of the case (1), centered at the head mid-point, identified as point#2 in the NORMA phantom (11). During this irradiation, the pairs of TLD-600 and TLD-700 were located inside the phantom holes (see Section *Thermoluminescent Dosimeters*).

### 2.2 Bonner Sphere Spectrometry

**Figure 2:**
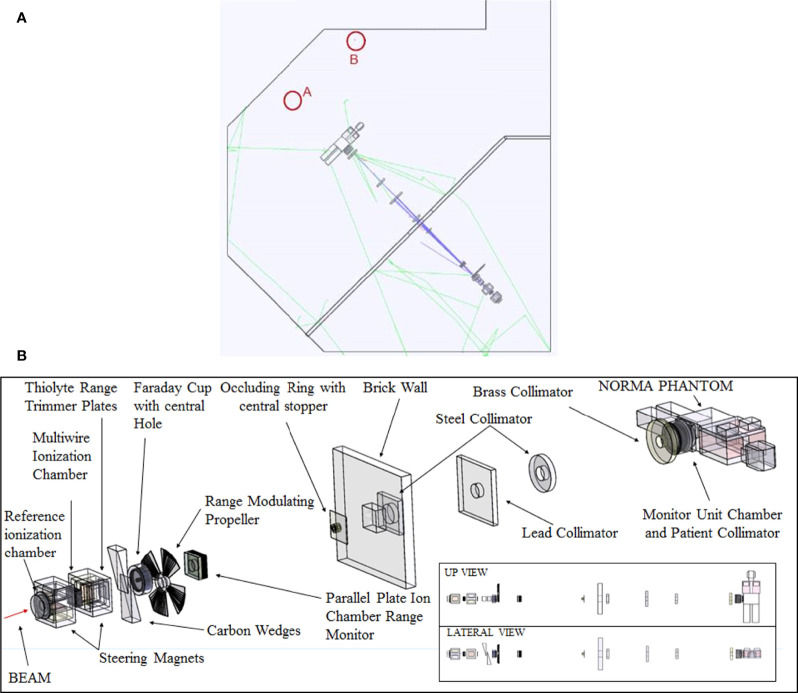
**(A)** Sketch of the treatment room with the points selected for BSS experimental measurements and MC simulations. Point A is located in the beam direction, downstream 2.33 m after the isocenter. Point B is 3.4 m away from the isocenter ~60° with respect to the beam line, downstream and to the right side. **(B)** Beam elements simulated in MC together with the NORMA phantom inside the treatment room. Room walls are not shown for clarity.

The active ERBSS from Universitat Autònoma de Barcelona (18) was used for the measurements of neutron spectra inside the treatment room. A proportional ^3^He counter (EURYSYS model 05NH1) was placed in the center of the moderating spheres. A set of 9 polyethylene spheres, with diameters 2.5, 3, 4.2, 5, 6, 7, 8, 10, and 12 in., was used. The 7 in. sphere was used, in addition, to host a 1 in. thick Cu inset and another 1 in. thick Pb inset to make it sensitive to high-energy (>20 MeV) neutrons. A discriminator level conveniently set in the counter electronics is employed for neutron-gamma separation. Measurements were performed at two points in the irradiation room, marked with A and B in **Figure 2A**, during the irradiation of the pelvic region of NORMA. Point A is located downstream in the beam direction, 2.33 m after the isocenter. As high-energy neutrons are mainly produced in the forward direction, the detectors placed at point A are able to detect high-energy neutrons coming from beam passive elements and a phantom. This position has been extensively evaluated in spot-scanning proton beams, such as the study of Mares et al. (2016) (19). Point B is 3.4 m away from the isocenter, in a direction ~60° with respect to the beam line, downstream and to the right side. Point B was selected close to the wall in a position where any other neutron monitor, such as a Berthold or a Tissue-Equivalent Proportional Counter (TEPC), could be located without interfering with the clinical routine. The knowledge of the neutron spectra for this kind of neutron monitors could be useful. No protons that could entangle the measurements are expected to reach points A and B. In fact, point A is behind NORMA, which is sufficiently thick to stop the primary proton beam, and point B is out of the primary beam direction. The necessary unfolding procedure for obtaining neutron spectra from Bonner sphere measurements was performed using the Frascati Unfolding Interactive Tool (FRUIT) unfolding code (20). When used in the *parametric mode*, Frascati Unfolding Interactive Tool (FRUIT) does not need a specified guess spectrum; it models the neutron spectrum using a reduced (≤7) set of meaningful physical parameters that depend on the type of radiation environment under study. The accepted solution is the spectrum obtained from the specific parameter array that fulfills better the unfolding convergence criteria. FRUIT can also be used in *numeric mode*, by perturbing an initial default *guess* spectrum according to the special gradient method (SGM). The *guess* spectrum is often obtained from computer simulation, but there are situations where the simulation results for a given energy range may be inaccurate or display significant uncertainties because of poor statistics, which may require considerable computing time. In such cases, it is particularly suitable to combine simulation results in the energy region where they are robust enough, with the spectrum obtained from FRUIT in the parametric mode in the region where simulation results may be poor. In both *parametric* and *numeric* modes, once a solution is accepted, uncertainties at each individual energy bin of the resulting fluence spectrum are evaluated from a variability analysis, either of the spectrum parameters (in parametric mode) or of a set of spectra obtained by randomly perturbing the solution (in numerical mode). These uncertainties are strongly energy and problem dependent. In this work, each spectrum obtained by *parametric mode* was used as the *guess* spectrum for a subsequent *numeric* unfolding to refine the solution. In such a way, the experimental results obtained from the unfolding process do not depend on the MC simulation, as would be the case if unfolding was performed starting from a simulated spectrum. The MC codes for neutron transport are known to be accurate up to 20 MeV, where the relevant cross sections are well known and evaluated from experimental results, but they provide model-dependent results above this energy. Cross sections up to 150 MeV are available only for the interaction of neutrons with only a few nuclear species, and physical models must be used otherwise (always above 150 MeV). The decision of using the described *parametric* + *numeric* approach was taken after the results of test runs using the MC spectrum as a guess either did not fulfill the convergence criterion or gave unphysical trends in the resulting spectra. The relevant dosimetric quantities and fractions of fluence and ambient dose equivalent for specified energy intervals, as well as their distributions, were obtained from the unfolding procedure and uncertainty analysis.

The global uncertainties of the total fluence and ambient-dose equivalent are normally within the range of 3%–6% and 4%–7%, respectively (18, 21).

### 2.3 Thermoluminescent Dosimeters

Standard 6 LiF/7 LiF pairs of dosimeters TLD-600/TLD-700 (3 × 3 × 0.9 mm^3^ chips) were used as an independent system to assess the thermal neutron fluences in the selected points inside the phantom. The sensitivity of both TLDs for photons can be considered the same because the chemical composition governs them, so either of them can be used for direct estimation of the photon-absorbed dose (22). In this work, absorbed doses were directly estimated from TLD-700. TLD-600 and TLD-700 had been previously calibrated using a ^137^Cs source at the Metrology Laboratory for Ionizing Radiation of the CIEMAT (see values below).

Neutron thermal fluences were obtained using the differences between TLD-600 and TLD-700 results for each measured point in NORMA (R) and the calibration factors as follows:


(1) $${\text{Φ}}_{th}=f_{600/700}^{n}\left[R_{600}-\frac{f_{700}^{\gamma }}{f_{600}^{\gamma }}R_{700}\right]$$

where the calibration factors used were $f_{600/700}^{n}=488$ n cm^-2^ (6%) for neutrons and $f_{700}^{\gamma }=1.86\times 10^{-4}$ mGy au^-1^ (4%) and $f_{600}^{\gamma }=1.99\times 10^{-4}$ mGy au^-1^ (5%) for gammas (11).

Neutron calibration was carried out at Physikalisch-Technische Bundesanstalt in scattered neutron reference radiation fields produced by a bare ^252^Cf and a D_2_O-moderated ^252^Cf neutron source (23).

TLD readouts were carried out using a Harshaw reader, model 4000, with linear heating from room temperature up to 280°C at a heating rate of 3°C s^−1^. A pre-irradiation thermal treatment of 1 h at 400°C, followed by a reproducible cooling down to room temperature, was constantly employed before reusing the detectors. The temperature and duration of the heating and cooling stages were adequately controlled.

The uncertainties of TLD results were derived from the standard uncertainties of calibration factors.

### 2.4 Monte Carlo Simulation

Computer simulations were carried out using the GAMOS (v 6.2)/GEANT 4 package (v 10.6) (24, 25) following a “full Monte Carlo” approach, that is, in a single run simulating the 201.36 MeV primary proton beam impinging in all elements present in the line (shutters, diaphragms, energy degraders, filters) close to the irradiation room, as well as the room walls and the presence of the anthropomorphic phantom (see **Figure 2B**). Calculations were done using two high-performance computer clusters at CIEMAT.

The aim of the simulation was to evaluate the neutron energy distribution inside the treatment room and the neutron spectra inside the NORMA phantom. To do this, a spherical detector (1 cm radius) was defined in each position and the neutron track length over the sphere volume was scored for each energy bin. In order to obtain the most realistic neutron spectra, the most up-to-date accurate geometry and material composition were considered. The Geant4 physics list used was QGSP_BIC_All_HP, recommended for proton and neutron transport under 200 MeV (26). The number of source protons was 1.9 × 10^9^ and 2.1856 × 10^10^ for the simulation inside the treatment room and the phantom, respectively.

A standard uncertainty from simulation has a statistical component associated to the number of source particles. However, simulations in this energy range rely on nuclear models and different results can be obtained when using different models or even codes (27). This variability could be used as a measure of the accuracy of simulations, and, based on results from De-Saint Hubert et al. (27), we estimate a value of 20% to combine to statistical uncertainty.

The ERBSS results from irradiation during the pelvic treatment, obtained independently from the MC simulation, served as the validation of the MC model.

### 2.5 Evaluation of Total Equivalent Dose in Organs

The total equivalent dose in organs was obtained as the average of the total dose equivalent in the representative points inside the phantom, using the assignment in **Table 2**. This section describes the methodology followed for evaluating the dose equivalent in each point.

**Table 2:** Point assignment for organ definition (11).

Organ	NORMA points
Thyroid	4
Esophagus	4, 9, 16
Lung	7, 8
Breast	5, 6, 15
Stomach	9, 11, 16
Liver	9, 10, 11, 16
Colon	11, 12
Urinary bladder	10
Ovary	11, 12
Prostate	11, 12
Uterus	11, 12

The total dose equivalent is calculated from the addition of the photon and neutron dose equivalent (*H_γ_* + *H_n_*). The photon contribution is directly calculated from the absorbed dose measured with the TLD-700 (taking into account that *w_R_* =1). The neutron dose equivalent can be derived using the following equation, as discussed in Romero-Expósito et al. (28):


(2) $$H_{n}=\text{Φ}\int_{E}Q\left(E\right)\cdot k(E)\cdot \frac{d\varphi_{i}\left(E\right)}{dE}\cdot dE$$

where Φ is the total neutron fluence, Q(E) is neutron quality factor as a function of energy, k(E) is the kerma factor for soft tissue [defined by the International Commission on Radiation Units and Measurements (ICRU)] as a function of energy (obtained from Siebert and Schuhmacher (29) for neutrons up to 20 MeV and in the work of Chadwick et al. (30) up to 150 MeV), and (dφ_i_(E))/dE, the energy spectrum of the unit neutron fluence at point *i*. This expression is based on the kerma approximation for the calculation of the absorbed dose, which is subsequently converted to dose equivalent by means of the quality factor Q(E).

In practice, to calculate *H_n_* at each point inside the phantom from equation (2), MC simulations are used in this work to determine the neutron energy spectrum (dφ_i_(E))/dE) at the relevant point. *Q*(*E*)·*k*(*E*) were taken from references (29) and (30). Finally, the total neutron fluence at the point (Φ) was calculated from the fluence measured using the thermoluminescence dosimeters. Then, at each point, the total neutron fluence equals the ratio of the thermal fluence Φ*_th_* (measured by TLD-600/700 pairs) to the fraction of thermal neutrons p*_th_* (obtained from the normalized simulated spectrum), as written in Equation 3:


(3) $$\text{Φ}=\frac{\Phi_{th}}{{\text{p}}_{th}}$$

Standard uncertainty in dose equivalent was obtained combining the experimental uncertainty of TLD results together with MC uncertainty. As equivalent dose in the organ is obtained as an average of the dose equivalent in several points, the uncertainty was derived as a propagation of uncertainty in each point.

## 3 Results

### 3.1 Neutron Field Inside the Treatment Room

**Figure 3:**
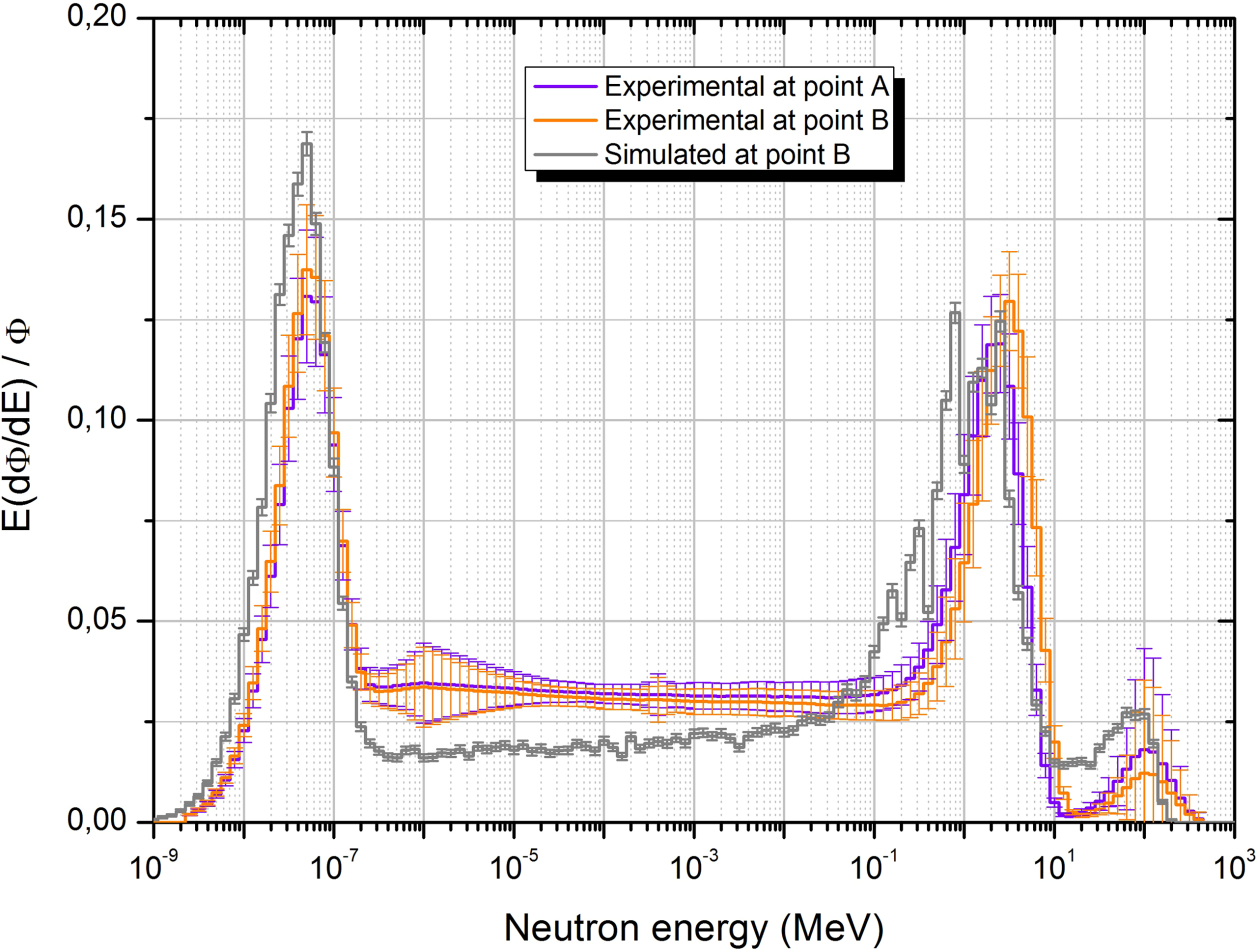
Neutron unit spectra obtained from unfolding with FRUIT at points A and B together with the simulation spectrum at point B.

The simulated neutron unit spectrum at point B is represented in **Figure 3** together with those obtained from unfolding with FRUIT the Bonner sphere measurements at points A and B. All spectra show in general similar trends, with prominent thermal (E < 0.4 eV) and fast (evaporation − 0.1 MeV < *E* < 20 MeV) peaks and a smaller contribution of high-energy neutrons (20 MeV < *E* < 200 MeV). The simulated spectrum displays a kind of double-peak structure in the evaporation region, approximately 1 MeV, which does not appear in the unfolded spectra. The origin of this discrepancy is that the energy binning of the simulation is fine enough to somehow preserve the resonances of the interaction cross sections of neutrons with heavy elements, while the energy resolution of Bonner sphere spectrometry is not enough to display this fine structure. In fact, Bonner sphere spectrometry has the advantage of being able to cover a huge energy interval (11 orders of magnitude) but with limited energy resolution. The evaporation peak for the simulated spectrum is widened toward the lower energies (0.1 MeV), but the fluence fractions corresponding to this peak are similar in all cases, as discussed later (**Table 3**). We consider that there is good agreement between simulated and unfolded spectrum in point B, especially from 0.1 MeV onwards, which is the relevant part in terms of the dose (see below) and because the total fraction of fluence above 0.1 MeV is similar for all spectra.

**Table 3:** Neutron fluence, ambient dose equivalent, fluence-averaged energy, dose equivalent-averaged energy, and average fluence to ambient dose equivalent conversion coefficient per unit proton dose imparted at the points of measurement inside the treatment room, as well as fluence and dose equivalent fractions for the different energy ranges from experimental determination and simulation.

		Point A experimental	Point B experimental	Point B simulation
Φ (cm^-2^ Gy^-1^)		(1.489 ± 0.055) × 10^6^	(1.577 ± 0.063) × 10^6^	
H* (10) (µSv Gy^-1^)		184 ± 12	203 ± 13	
E_Φ_ (MeV)		3.90	3.10	
E_H*_ (MeV)		9.66	7.70	
h* (10) (pSv·cm^2^)		123.7 ± 6.8	129.8 ± 6.6	
Fluence fractions	E ≤ 0.4 eV	30.9%	31.8%	36.1%
	0.4 eV < E < 100 keV	37.1%	35.5%	24.2%
	100 keV ≤ E ≤ 20 MeV	29.1%	30.6%	35.5%
	E > 20 MeV	2.9%	2.0%	4.2%
H* (10) fractions	E ≤ 0.4 eV	3.0%	2.9%	2.8%
	0.4 eV < E < 100 keV	4.1%	3.8%	2.6%
	100 keV ≤ E ≤ 20 MeV	85.3%	88.4%	83.9%
	E > 20 MeV	7.6%	4.9%	10.7%

**Figure 4:**
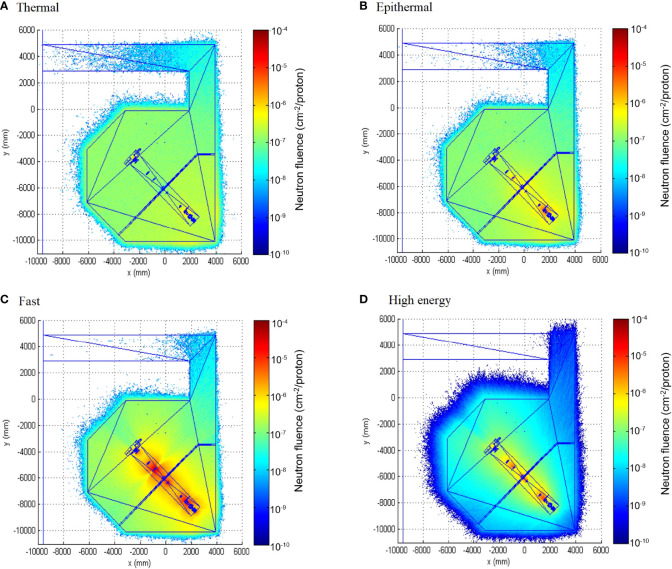
Neutron fluence map inside the facility for the four relevant groups: thermal (E < 0.4 eV) (**A**), epithermal (0.4 eV < E < 0.1 MeV) (**B**), fast (0.1 MeV< E < 20 MeV) (**C**) and high energy (20 MeV < E < 200 MeV) (**D**).

The fact that the spectra in the two points are similar (see also fluence fractions in **Table 3**) is a consequence of the particular geometric characteristics of the beam line, beam elements, and irradiation room. In fact, all beam elements relevant for fast and high-energy neutron production are located quite far away from the treatment place, outside the irradiation room and without shielding, so that even the forward-scattered neutrons almost uniformly cover the irradiation room. This behavior is clearly represented in **Figure 4**, where the spatial distribution of neutron fluence simulated in the four relevant energy groups is displayed. From this figure, we can conclude that high-energy neutrons, originated almost exclusively in the beam elements, are highly directional (even outside the irradiation room), while thermal neutrons almost uniformly fill the irradiation room volume. The behavior for fast and epithermal neutrons (0.4 eV < *E* < 0.1 MeV) is halfway the others, with a significant amount of fast neutrons but a smaller amount of epithermal neutrons, also being produced in the beam elements and decreasing their directionality for smaller energies. The fact that the epithermal component is higher in the unfolded spectra than in the simulated one is explained by the impossibility to introduce in the simulation the detailed geometry and composition of all elements, structures, instruments, and other stuff present in the irradiation room and around the beam line, which contribute to the thermalization of neutrons.

Neutron fluence (Φ), ambient dose equivalent (H^*^10), fluence-averaged energy (E_Φ_), dose equivalent-averaged energy (E_H*_), and average fluence to ambient dose-equivalent conversion coefficient (h*10) at points A and B per unit proton dose imparted are presented in **Table 3**. While neutron fluence is of the order of 10^6^ cm^-2^/Gy, H* (10) is of the order of 0.2 mSv/Gy in the region away from the patient. A slightly higher amount of neutrons at the patient position would be expected given that it is closer to the beam elements. The fractions of fluence and ambient dose equivalent for the thermal, epithermal, fast, and high-energy components of the neutron field are also displayed in the table. Note that the most important contribution to the dose is that from the evaporation region due to the strong energy dependence of the fluence-to-dose-equivalent conversion coefficients. It is worth noting that, even if the visual aspect of the simulated and the unfolded spectra at point B is not the same, the fractional contribution to the fluence and ambient dose equivalent of neutrons in the different energy intervals considered differ only in a few percentage points. In fact, the biggest discrepancy (approximately 11% difference), from 24.2% of the total fluence in the simulated spectrum to 35.5% of the total fluence in the experimental one, is found in the contribution to epithermal (0.4 eV < E < 100 keV) fluence. Discrepancies are 4.3% of the total fluence in the thermal (E ≤ 0.4 eV) component, 5.1% in the fast (100 keV ≤ E ≤ 20 MeV) component, and 2.2% in the high-energy (E > 20 MeV) component. The contribution to the total ambient dose equivalent of the thermal and epithermal components is small, and the total contribution to the dose of the fast + high-energy components (those that are relevant because the fluence-to-dose-equivalent conversion coefficients) represents 94.6% for the simulated spectrum and 93.3% for the experimental one.

The FRUIT-unfolding process (20) leads to uncertainties of approximately 3% in fluence determination and 7% in H* (10) determination. The sources of this uncertainty, given at the standard level (*k* = 1), are as follows:

- Counting statistics in the detectors at the center of the Bonner spheres (type A): approximately 1% in this experiment
- The uncertainty of the sphere’s response matrix, obtained from simulation and experimental validation (type B): 3% average.

In addition, uncertainties at each individual energy bin of the resulting fluence spectrum are obtained from a variability analysis, either of the spectrum parameters (in parametric mode) or of all spectra that fulfill the convergence criteria during unfolding (in numerical mode). These uncertainties are strongly energy and problem dependent. In our case, they range from 7% in the epithermal to fast region to over 100% in the high-energy region, as seen in **Figure 3**. Uncertainty bars at the experimental spectra in **Figure 3** include all these uncertainty sources, but only statistical simulation uncertainties are displayed in the simulated spectrum. The systematic uncertainty related to the physics models used inside the MC code could rise up to 20%, as discussed by De-Saint Hubert et al. (28), especially in the high-energy region.

### 3.2 Neutron Field Inside the Phantom

**Figure 5:**
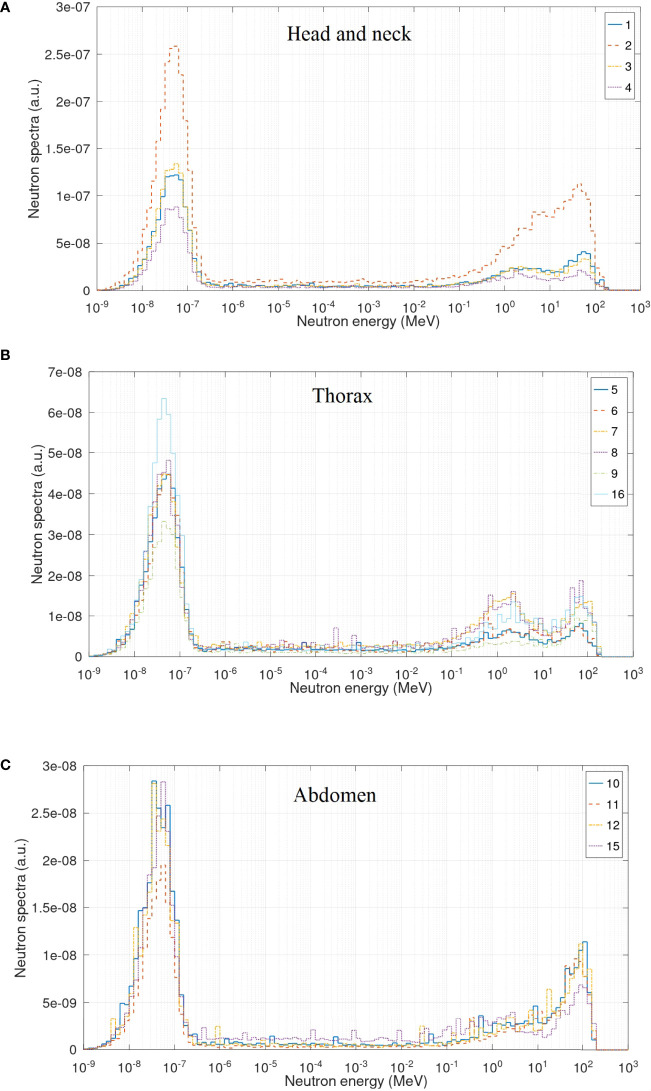
Neutron spectra inside the NORMA phantom in the head and neck **(A)**, thorax **(B)**, and abdomen **(C)** region.

**Figure 5** shows the neutron spectra inside the 16 points in NORMA phantom. Statistical uncertainties from the MC simulation were 8% on average. There are several differences with spectra inside the room. The most prominent one is the important reduction in the fast neutron peak. This fluence attenuates as neutrons go through the tissue and, consistently, the fast neutron fraction becomes lower, from approximately 30% in the room to 16% on average in the phantom (see fluence fractions in **Table 4**). This fast neutron attenuation leads to an increase of the thermal neutron peak. In terms of fluence fractions, from approximately 31% in the room to 56% on average inside the phantom. In **Figure 5B**, the points with a higher fast peak are those corresponding to lung tissue, which have lower density and, therefore, less attenuation.

**Table 4:** Quantities evaluated in the points inside the phantom.

Point	MC neutron fluence fractions				TLD results	
	Thermal (%)	Epithermal (%)	Fast (%)	High energy (%)	Thermal neutron fluence per treatment gray (×10^6^ cm^-2^ Gy^-1^)	Photon dose equivalent per treatment gray (×10^-2^ mSv/Gy)
1	54	14	18	14	6.29 ± 0.38	9.23 ± 0.37
2	48	12	22	19	*	*
3	57	14	17	12	8.27 ± 0.50	12.09 ± 0.48
4	56	15	18	11	9.47 ± 0.57	9.23 ± 0.37
5	61	16	14	9	5.34 ± 0.32	7.19 ± 0.29
6	58	16	16	9	6.05 ± 0.36	7.40 ± 0.30
7	47	17	23	13	5.53 ± 0.33	11.64 ± 0.47
8	47	18	22	14	5.61 ± 0.34	10.52 ± 0.42
9	58	15	12	15	4.67 ± 0.28	5.12 ± 0.20
10	59	9	12	20	2.82 ± 0.17	3.73 ± 0.15
11	54	7	14	25	1.320 ± 0.079	3.71 ± 0.15
12	58	9	12	21	2.32 ± 0.14	4.21 ± 0.17
13	61	10	13	15	2.86 ± 0.17	3.42 ± 0.14
14	60	10	14	16	3.19 ± 0.19	4.30 ± 0.17
15	55	18	14	13	3.71 ± 0.22	3.46 ± 0.14
16	57	13	17	13	7.30 ± 0.44	10.22 ± 0.41

*No TLD was inserted in the isocenter of the treatment.

**Table 4** also presents the results of the photon dose equivalent and neutron thermal fluence measured with TLD dosimeters. While photon doses range between 0.121 and 0.0342 mSv/Gy, neutron thermal neutron fluences range from 9.47 × 10^6^ to 1.32 × 10^6^ cm^-2^/Gy. The general trend of both quantities is to decrease as the distance to the isocenter increases, with fluctuations associated to the depth of the point in the phantom.

Neutron dose equivalents were calculated from data in **Table 4** using equations 2 and 3. These values range from 1.22 to 0.237 mSv/Gy. Uncertainties of these values were 31% on average, and were composed of

- Statistical uncertainty of the MC simulation: 8% (type A)
- Experimental uncertainty of the TLD measurements: 15% on average, position dependent (type A)
- Systematic uncertainty related to the physics models used inside the MC code, as discussed by De-Saint Hubert et al. (28): 20% (type B).

All uncertainties were calculated at the standard (*k* = 1) level.

**Figure 6:**
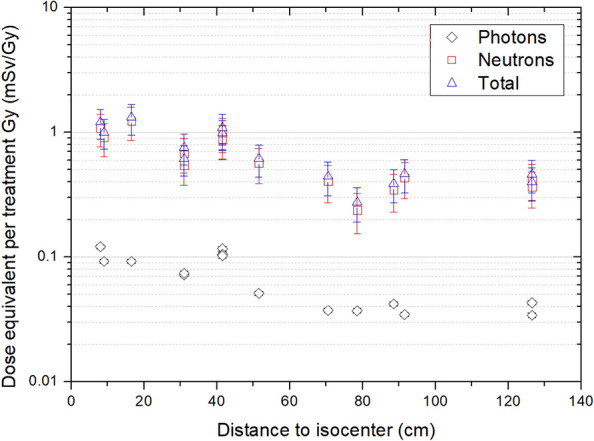
Photon, neutron, and total dose equivalent per treatment Gy as a function of the distance to the isocenter.

**Figure 7:**
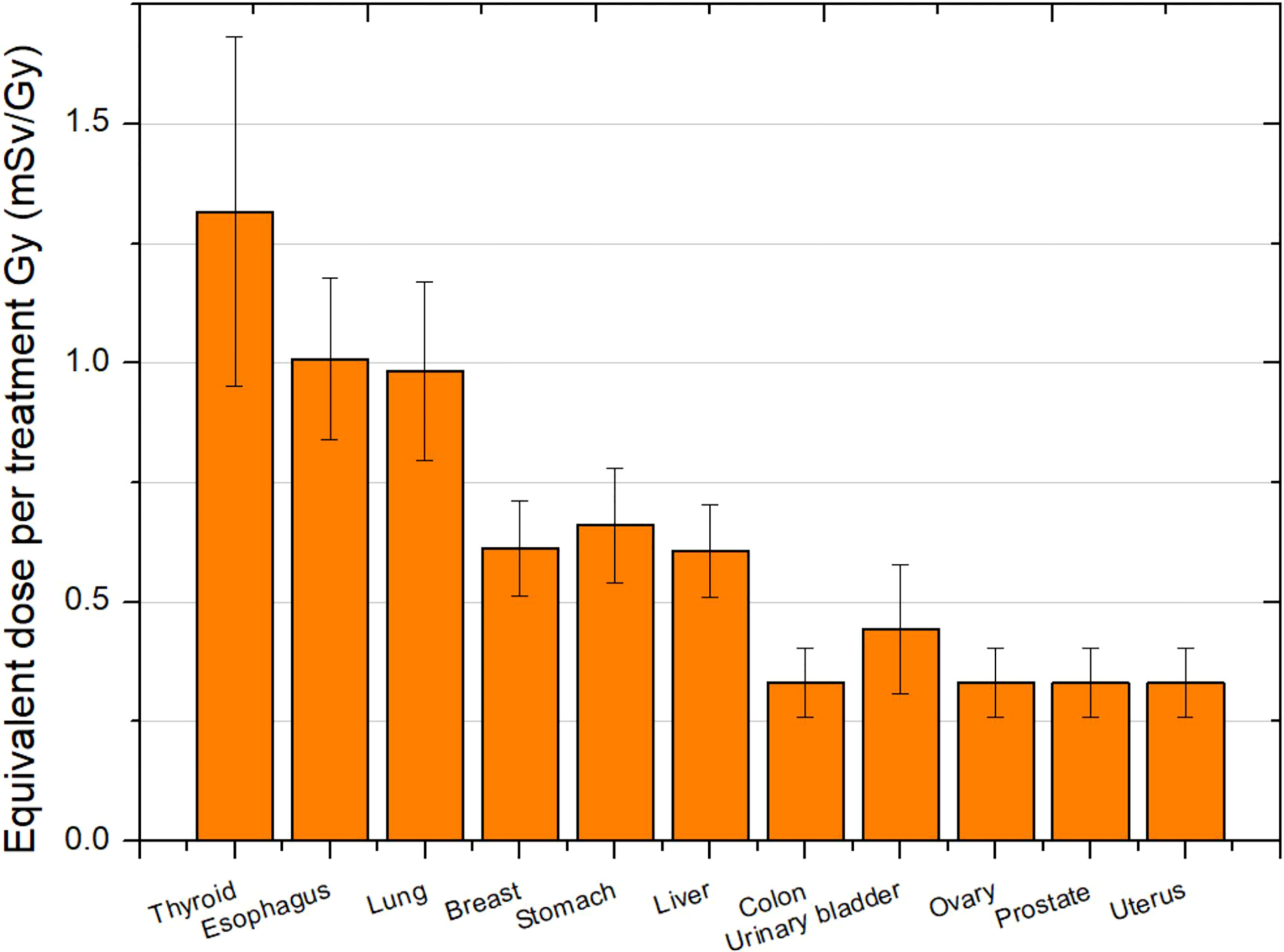
Total equivalent dose in organs per treatment Gy for a head treatment.

**Figure 6** shows the photon, neutron, and total dose equivalent as a function of the distance to the isocenter in the craneo-caudal direction. As can be clearly noticed in the figure, the contribution of photons in this irradiation is very low. Photons represent on average 10% of the total dose. From these values, using the assignment in **Table 2**, the equivalent dose in organs was calculated. Values are presented in **Figure 7**. Equivalent doses keep the same trend of reducing when going farther from the target. For thyroid, the closest organ to the target, the equivalent dose is 1.32 mSv/Gy. In abdomen, for example, the stomach has an equivalent dose of 0.661 mSv/Gy, and in the pelvic area, ovaries present 0.331 mSv/Gy. The uncertainties of equivalent dose range from 16% to 30%.

## 4 Discussion

This work presents a methodology for the evaluation of total equivalent doses in organs based on measurements complemented by the MC simulation. Simulation is essential for neutron dosimetry as the biological effect of neutrons depends on their energy and, currently, simulation is the only way to evaluate the wide range of neutron energies inside a patient. However, simulations, in turn, must be validated against measurements. In our case, ERBSS was used to determine, with a procedure completely independent from simulation, the neutron energy distribution at two points in the treatment room. The fluence and ambient dose equivalent per treatment Gy, as well as percent contribution to the fluence and ambient dose equivalent of neutrons in four energy groups, were also obtained from ERBSS measurements. Finally, the ERBSS results were used to validate the simulation results in one of the points. Although simulated and experimental spectra are not identical, probably due both to the constraints of the parametric unfolding process used and to not being able to simulate all elements affecting neutron production, their general trend, and the fractional contributions to fluence and the ambient dose equivalent agree within a few percentage points, as can be seen in **Table 3**. The biggest discrepancy (approximately 11% difference) is found in the contribution to epithermal (0.4 eV < E < 100 keV) fluence, which gives approximately 35% of the total experimental fluence and approximately 24% of the simulated one. This is consistent with incomplete simulation of elements contributing to the energy degradation of neutrons. Nevertheless, given the strong energy dependence of the fluence to the ambient dose equivalent conversion coefficient h* (10), the contribution of this epithermal component to the ambient dose equivalent H* (10) is very small. Once MC simulations are validated, the neutron spectra inside the patient can be calculated and subsequently used for dose equivalent determination using Equation 2. The approach followed in this work was to evaluate the total neutron fluence appearing at the equation using an independent neutron dosimeter. With sufficient knowledge of the accelerator parameters, it is also possible to evaluate this fluence merely from simulation, in which case an additional validation for the fluence value would be needed. Regardless of the circumstances, the methodology can be reproduced using any other phantom in any other facility.

Regarding our particular results, the spectra measured inside the treatment room are similar to those reported by Howell et al. (31) for neutrons with energies higher than a few eV in the passive scattering system Mevion 250, that is, a high contribution of evaporation neutrons and a lower peak of high-energy neutrons. The contribution of thermal neutrons is related to the treatment room volume and, therefore, is highly facility dependent. Results from Howell et al. (31) show a smaller proportion of thermal neutrons than ours, for instance. Nevertheless, their contribution to the ambient dose equivalent is very low, and then, the room size does not represent an important parameter to take into account when analyzing the neutron production. The most relevant parameters are the proton energy, target volume (trough field size and Spread-Out Bragg Peak (SOBP) width), and the distance to neutron source. The latter is not simple to define in passive facilities, as several beam elements contribute to neutron production (32). However, the isocenter is usually considered as a reference for the position inside the room. In the case of Howell et al. (31), they reported an H*(10) value 3.90 mSv/Gy at 50 cm from the isocenter. This value is approximately 17 times larger than ours, which can be explained by their higher proton energy (250 MeV), the bigger target volume (a whole brain treatment), and the smaller distance from the point of measurement to the patient location (isocenter). Our results show a better agreement with Zheng et al. (33) and Han et al. (34) (see values in **Table 5**), where the energy and the distances to the isocenter were similar to ours. We could conclude that away from the patient (more than 2 m from the isocenter) H*(10) is of the order of 0.1 mSv/Gy. The dose range obtained for these passive scanning facilities differs from that obtained in scanned beam facilities, where the ambient dose equivalent is of the order of µSv/Gy in the area away from the patient (19).

**Table 5:** Comparison of H* (10) with other works.

Reference	Method	Proton energy (MeV)	Beam characteristics	Distance to isocenter (cm)	Angle with beam axis (°)	H* (10) mSv/Gy
Zheng et al. (28)	MC	250	Unmodulated, 10 × 10 cm^2^ aperture	200	0	0.18
Howell et al. (26)	ERBSS	250	17 cm range, 16 cm modulation, 13 cm diameter aperture	50	90	3.9
Han et al. (29)	WENDI-II	218	21 cm range, 5 cm modulation, 8 cm diameter aperture	200	0	0.313
				283	45	0.203
Our work	ERBSS	200	10.5 cm range, 3 cm modulation, 3 cm diameter aperture	233	0	0.209
				340	60	0.235

Ambient dose equivalent is given the symbol H*(10).

A noteworthy aspect of our results is that we obtained similar values of H*(10) in both measurement positions, showing a quite-uniform exposure to external neutrons around the patient location. Point A showed a lower value, due to a slight attenuation in the phantom, which can be noticed in **Figures 4C, D**. This result is a geometry effect also showed by Moyer et al. (32). Their MC simulation allowed to conclude that only a small fraction of the neutrons generated in the scatters of the beamline reaches the patient. Most of the neutrons that do reach the patient are generated in the precollimators, the patient-specific aperture, and within the patient themselves. In addition, Howell et al. (31) tested the same treatment with no phantom and with different phantom materials (water, soft tissue, and plastic water). They found a nearly negligible difference in the fluence for the different phantom scenarios; the differences between no phantom at the isocenter and either water or soft tissue phantom were <2%. This indicates that at 50 cm from the isocenter (approximately 25 cm from the proximal surface of the phantom) for the considered fields, there was essentially no contribution from the neutrons produced inside the patient. That is, internal neutrons made a low-to-negligible contribution to the neutron dose equivalent in passive beam lines. Their effect is relevant in closest region around the target volume.

The low contribution of internal neutrons in passive beam lines explains the slow decay of the neutron dose equivalent inside the phantom as the distance to the isocenter increases. Our results showed a reduction from 1.22 mSv/Gy at 17 cm to 0.346 mSv/Gy at 89 cm from the isocenter. Taking into account that the locations inside NORMA are at different depths and the effect of lung tissue at middle distances, our values may not be completely representative of the trend with distance but are consistent with those reported in Hägl et al. (35). In that work, the neutron dose equivalent was measured inside the Alderson−Rando phantom in a double- scattering beamline using CR-39 detectors located along the medial patient axis during a prostate treatment. The neutron equivalent dose was 1 and 0.1 mSv/Gy at approximately 12 and 78 cm, respectively. By contrast, in the scanned beam, the dose-equivalent reduction can be of almost two orders of magnitude from positions close to the isocenter (1 mSv/Gy) to positions up to 20 cm from the isocenter (0.01 mSv/Gy) (36).

Finally, our equivalent doses in an organ can be compared to those reported by Farah et al. (37). In that work, a standard intracranial treatment with a 178 MeV proton beam was simulated with Monte Carlo N-Particle eXtended (MCNPX) code in a computational phantom. For the thyroid, the closest organ to target, they found an equivalent dose of 1.5 mSv/Gy, while in our case, it was 1.32 mSv/Gy. These values are compatible within our uncertainties, although a higher value would be expected in our case as we considered a higher proton energy. However, the small volume of our target could be a reason of obtaining a lower value. For the rest of organs, as expected because of the proton energy, we found systematically higher doses. For example, while Farah et al. (37) reported an equivalent dose of 0.63, 0.39, and 0.43 mSv/Gy in the lung, stomach, and liver, respectively, our results were 1.01, 0.661, and 0.606 mSv/Gy for the same organs. It must be taken into account that one important limitation of our work is that organs were defined using a few points in the NORMA phantom. However, the comparison with Farah et al. (37) show that the reported values are reliable within their uncertainties. An improvement would imply to modify the phantom, drilling a higher number of holes for placing the detectors, to perform a simulation at all these points, which would be more representative of the organs of interest, and to be able to perform measurements in a bigger number of points, more representative of the organs of interest. In this way, the methodology presented could be reproduced, and the use of a soft tissue phantom with a high amount of detector holes could lead to more accurate estimations.

Results could allow to conclude that the equivalent dose in organs could be of the order of 0.1 or 1 mSv/Gy in passive facilities. It is worth noticing that these values represent both neutron and photon contributions. However, photons represent only 10% of the value, and therefore, neutrons require major attention in passive facilities.

## Data Availability Statement

The raw data supporting the conclusions of this article will be made available by the authors, without undue reservation.

## Author Contributions

FS-D: Leader of the project, expert in medical physics, participated in the design and realization of the experiments. CD: Expert in neutron dosimetry and responsible for the experimental neutron spectrometry. JL: Expert in Monte Carlo and responsible for the simulation. MR-E: Expert in neutron dosimetry and out-of-field dosimetry in radiotherapy, participated in the design and realization of the experiments and data analysis, and wrote the first draft of the manuscript. BS-N: Expert in medical physics, radiobiology, and peripheral dose in radiotherapy, participated in the design and data analysis, in particular, in the evaluation of the organ dose from measurements in the phantom. JN-C: Local member, participated in the design and realization of the experiments. JT: Expert in medical physics and dosimetry, participated in the experiments. LI: Expert in medical physics and dosimetry, participated in data analysis. AD: Expert in medical physics and out-of-field dose in radiotherapy, participated in the coordination and improvement of the manuscript. All authors contributed to the article and approved the submitted version.

## Funding

This work has been partially carried out on the ACME cluster, which is owned by CIEMAT and funded by the Spanish Ministry of Economy and Competitiveness project CODEC2 (TIN2015-63562-R) with FEDER funds as well as supported by the CYTED-co-founded RICAP Network (517RT0529). MR-E acknowledges funding from Euratom’s research and innovation programme 2019-20 under grant agreement no. 945196. BS-N acknowledges project Fondecyt N1181133.

## Conflict of Interest

The authors declare that the research was conducted in the absence of any commercial or financial relationships that could be construed as a potential conflict of interest.

## Publisher’s Note

All claims expressed in this article are solely those of the authors and do not necessarily represent those of their affiliated organizations, or those of the publisher, the editors and the reviewers. Any product that may be evaluated in this article, or claim that may be made by its manufacturer, is not guaranteed or endorsed by the publisher.
